# Diversity and evolution of archaeal immune strategies

**DOI:** 10.1093/nar/gkag225

**Published:** 2026-03-14

**Authors:** Laura Martínez-Alvarez, Xu Peng

**Affiliations:** Department of Biology, University of Copenhagen, 2200 Copenhagen N, Denmark; Department of Biology, University of Copenhagen, 2200 Copenhagen N, Denmark

## Abstract

Archaeal antiviral defense systems remain poorly characterized despite recent advances in understanding prokaryotic immunity. Here, we analyze 7747 archaeal genomes, the largest and most diverse dataset to date, revealing a striking disparity in defense system prevalence and diversity compared to Bacteria. Nearly one-third of archaeal genomes have no detected systems beyond CRISPR-Cas and restriction-modification (in contrast to only 2.2% bacterial genomes), and only 50–55% contain CRISPR-Cas systems, far below previous estimates. Many known defense systems appear restricted to Bacteria, while several single-gene putative candidate systems (PDCs) recently identified through a guilt-by-embedding approach are enriched in Archaea. Phylogenetic analyses suggest that PDC-S70 and PDC-M05 likely originated in Archaea, representing rare archaeal contributions to the prokaryotic immune repertoire. Consistent with earlier studies, our findings support the existence of deep evolutionary links between archaeal and eukaryotic systems for argonautes and viperins. These analyses highlight both the underexplored nature and the evolutionary significance of archaeal immunity, calling for expanded efforts to uncover archaeal-specific systems and improve our understanding of immune evolution across domains of life.

## Graphical Abstract

**Graphical Abstract:**
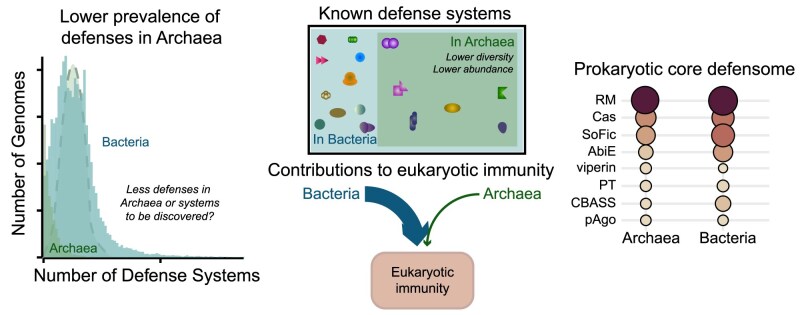

## Introduction

Mobile genetic elements (MGEs) are major drivers of horizontal gene transfer (HGT). They contribute to microbial genetic diversity and evolutionary innovation by facilitating the spread of adaptive traits such as antibiotic resistance and virulence factors through mechanisms such as transformation, transduction, and conjugation [1, 2]. Their interactions with hosts range from mutualistic to parasitic, often imposing significant fitness costs [3, 4].

To counteract the costs imposed by MGEs, prokaryotes have evolved diverse defense strategies, from receptor modifications to sophisticated defense systems that degrade or modify nucleic acids, arrest cell growth, or disrupt membranes [5, 7]. Defense systems often cluster in genomic “defense islands”, frequently alongside MGEs [8]. These are regions prone to HGT, an arrangement that may facilitate their synergy and co-mobilization [9, 10]. The reciprocal selective pressure between MGEs and host defenses drives an ongoing evolutionary arms race, resulting in rapid innovation of prokaryotic immunity [4].

Over 300 antiviral defense systems have been identified to date, many through recent advances in computational approaches [11–15]. Although more than one-third of known systems also appear in Archaea [6], studies of archaeal immunity have remained limited in scope. Until recently, analyses relied primarily on RefSeq genomes, where archaea comprised fewer than 2% of the data [16, 17]. This limitation has led to assumptions that archaeal immune landscapes largely mirror those of bacteria, with CRISPR-Cas and restriction-modification (RM) systems being the primary known components.

Two recent studies expanded defense analyses in Archaea but focused primarily on the *Asgardarchaeota* phylum or on specific systems (viperins and argonautes) [18, 19]. Only CRISPR-Cas diversity has been comprehensively mapped across the domain [20–22]. Broader evaluations of other systems, including classical ones like restriction-modification, are lacking.

Meanwhile, archaeal genome availability has surged, especially through the recovery of uncultured lineages via environmental sequencing [23, 24], making a comprehensive re-evaluation of archaeal defenses timely. Understanding archaeal immunity is key to reconstructing the evolution of antiviral defense across living organisms, especially given the proposed archaeal ancestry of eukaryotes [25, 26] and evolutionary connections between bacterial and eukaryotic systems [27 -29].

In this study, we analyze 7747 archaeal genomes, the largest and most taxonomically diverse dataset to date, to reassess the distribution, diversity, and evolution of antiviral defense systems in Archaea.

## Materials and methods

### Data

The GTDB database (accessed on May 5, 2023) was used to retrieve the accessions and metadata for archaeal and bacterial genomes with over 50% completeness and < 20% contamination [24, 30]. From this, we obtained a dataset of 7747 archaeal genomes and 40 000 bacterial genomes (the latter randomly subsampled from the 394 933 available bacterial entries), which were downloaded from NCBI [31] to create the Archaea and Bacteria datasets, respectively (Supplementary Table 1). Proteomes for each genome were retrieved from NCBI when available, or predicted from the genomic sequence using Prodigal v2.6.3 [32]. Supplementary Fig. 1 depicts the taxonomic diversity of the genomes in the Archaea dataset and includes 10 additional 10 GTDB phyla not covered previously [6, 17].

### Identification of defense systems

To identify known defense systems in the archaeal and bacterial genomes, we used DefenseFinder v1.2.2 [17], Padloc v2.0.0 [16], and CRISPRCasTyper v1.8.0 [33] with default settings. These tools identify antiviral defense systems by detecting specific protein families through HMM-based homology searches and applying system-architecture rules to group hits into canonical defense operons. For identifying restriction-modification systems based on the REBASE repository (accessed in September 2024) [34], archaeal proteins were matched to the REBASE entries using MMseqs2 (release_15–6f452) [35] with parameters set to *–min-seq-id* 0.65, *–cov_mod* 0, and *–c* 0.8. Proteins were assigned functions and type according to the best match based on e-value scores, resulting in a pool of candidate components for restriction-modification systems. To refine these candidates, we excluded proteins identified as part of non-RM systems by Padloc or DefenseFinder. An in-house script was then used to retrieve the genomic neighborhood of all proteins annotated as restriction enzymes, capturing five genes upstream and five genes downstream of each restriction nucleases. This neighborhood analysis allowed the identification of RM systems meeting specific criteria: Type I RM – presence of type I R, M, and S components; Type II RM – presence of type II R and M components; Type III RM – presence of type III R and M components; Type IV – the presence of a type IV module alone; Type IIG – the presence of a type IIG module alone. A comprehensive list of predicted RM modules is available in Supplementary Table 6. Analyses and graphical visualization of data were carried out using R version 4.3.3 (2024-02-29) [36], RStudio version 2024.04.1 Build 748 [37], and the package *ggplot2* v3.4.2 [38]. The final comparison of the archaeal and bacterial immune landscapes was done using the output of Padloc after discarding entries labeled as DNA modification systems (DMS), which denote proteins involved in defense systems that modify DNA but cannot be classified as complete defense systems, and VSPR entries, which are not defense systems. For the identification of archaeal restriction-modification systems, the output of the REBASE-based approach described above was used instead of the PADLOC prediction.

### Statistical analysis

#### Taxonomic prevalence analysis

The prevalence of each defense system across archaea phyla was modelled using binomial generalized linear models. Several systems were rare within individual phyla, and ordinary logistic regression showed signs of data separation and infinite or poorly behaved standard errors. To address this, we used a bias-reduced logistic regression model with a logit link in the *brglm2* package (v0.9) [39], which provides finite and less biased estimates under separation. Estimated marginal means were calculated with the *emmeans* package (v1.11.1) [40] to compare phylum-specific prevalence against the overall archaeal mean. ρ-values were adjusted for multiple tests using the false discovery rate (FDR) method, with significance defined as adjusted *P* < 0.05 (Supplementary Table 9). Phyla with fewer than 10 genomes were not included in the analysis.

#### Abundance and diversity of defense systems across domains

Statistical analysis about the abundance and diversity of defense systems in the archaeal and bacterial datasets was done using the *vegan* package (v.4.1.3) [41]. The Shapiro–Wilk test showed evidence of non-normality for the genome size (W = 0.893, *P*-value < 2.2 × 10^−6^ for archaea and W = 0.968, *P*-value < 2.2 × 10^−6^ for bacteria), total system counts per genome (W = 0.678, *P*-value < 2.2 × 10^−6^ for archaea and W = 0.735, *P*-value < 2.2 × 10^−6^ for bacteria) and defense system diversity per genome (W = 0.788, *P*-value < 2.2 × 10^−6^ for archaea and W = 0.957, *P*-value < 2.2 × 10^−6^ for bacteria) distributions in the archaeal and bacterial datasets.

#### Effect of genome completeness on defense system abundance

Genome completeness estimates from GTDB metadata used for this analysis are provided in Supplementary Table 1. A filtered dataset of high-quality MAGs (≥90% completeness) was used to investigate the effect of assembly completeness on defense system prevalence for both Bacteria and Archaea (Fig. 2, right panel). To determine if prevalences were different between high-quality domain datasets, 95% confidence intervals (Wilson method) were calculated using the function *binom.confint()* from package *binom* (v1.1.1.1) [42]. For each core defense system, a logistic regression model was fitted with presence/absence as the response and genome completeness and domain as predictors using the function *glm()*. Predicted prevalence and Wald-type 95% confidence intervals were calculated by transforming model predictions from the logit scale to probabilities using the inverse logit function (Supplementary Fig. 6).

#### Contribution of genome size and domain to defense system abundance

Correlation between genome size and defense system abundance and diversity was evaluated using Spearman’s rank correlation. Linear regression models (defense system counts ∼ genome size) were fitted using the *lm()* function in R (R^2 ^= 0.218, F(1,47745) = 13 330, *P* < 0.001). Residuals were extracted using *residuals()* and compared between domains using a Wilcoxon rank-sum test. Variance partitioning was performed using *varpart()* function from the *vegan* package. Defense system counts were normalized by genome size (per Mb) to compare density across domains, and the results are shown in Supplementary Fig. 4.

#### Optimal growth temperature and archaeal core-defensome abundance

Optimal growth temperature (OGT) for archaeal genomes was estimated using Tome [43], which predicts growth temperature from proteome composition. Genomes were classified as mesophilic (25-≤50°C), thermophilic (≥ 50-<80°C), or hyperthermophilic (≥80°C). Differences in the core defensome abundance across temperature categories were first assessed using the Kruskal–Wallis test, followed by Dunn’s *post-hoc* test with the Bonferroni correction using the dunn.test package (v1.3.6) [44]. To quantify the relationship between OGT (continuous) and defensome abundance, we fitted negative binomial generalized linear models (NB-GLMs) with a logarithmic link using the MASS package (v7.3.60.22) [45] to account for overdispersion. For RM abundance, total defensome abundance, and system diversity per genome, zero-truncated hurdle negative binomial models were used to account for zero-deflation and overdispersion using the *pscl* package (v1.5.9) [46]. Nagelkerke’s pseudo-R^2^ of the negative binomial model was calculated using package *performance* (v0.15.3) [47]. Model selection and diagnostic checks were performed using the package DHARMa (v.0.4.7) [48]. Corresponding results are shown in Supplementary Fig. 8.

To quantify how individual genomic and ecological variables contribute to defense abundance variation in each domain, we applied the same NB-GLM framework described above. Separate one-predictor models were fitted with log-transformed genome size, phylum, genome completeness, or optimal growth temperature (Archaea only) as predictors, and Nagelkerke’s pseudo-R² was calculated for each predictor. A full model containing all predictors was also fitted to evaluate combined explanatory power (Supplementary Fig. 4E).

Analyses were performed in R (v4.4.0) [36]. All visualizations were generated using the *ggplot2* package (v3.4.2) [38]. Figures of composite graphs were generated using the package *patchwork* (v1.3.0) [49].

### Phylogenetic analyses

Components of defense systems identified by Padloc were used for all phylogenetic analyses, with restriction-modification systems, which were analyzed using components of the REBASE-based approach. For analysis of specific defense systems, concatenated DndC and DndD amino acid sequences were used for the DndABCDE phosphorothioation system, PIWI-domain components for argonautes, the AbiEii module for AbiE systems, the cyclase module for CBASS, and the M05A nucleotidyltransferase of PDC-M05. Eukaryotic sequences were obtained from the following sources: eukaryotic viperins from Shomar *et al.*[19], cGAS-like pattern recognition receptors (cGLRs) from Li *et al.*[50] and argonautes from Swarts *et al.* [51].

To reduce sequence redundancy, we clustered the components of each defense system using cd-hit v4.8.1 at a 65% similarity threshold [52]. Amino acid sequences were then aligned with MAFFT v7.505 using the *“auto”* option [53] and trimmed with TrimAl v1.5.rev0 [54] using the “*gappyout*” setting. Preliminary phylogenetic trees were constructed with FastTree v2.1.11[55] with parameters *–lg* and *–boot 100*. The preliminary trees were pruned for retaining sequence diversity using Treemer v.0.3 [56], resulting in a reduced dataset of sequences for constructing the phylogenetic trees presented in Fig. 5. For these trees, sequences were newly aligned using MAFFT with parameters *–maxiterate 1000* and *–localpair*, and then trimmed with TrimAl using the *-gt 0.3* option. Final phylogenetic trees were constructed with IQ-TREE v2.0.7[57] using options *–m MFP –bb 1000 –alrt 1000* and *-bnni* to select the best-fit model using the Model Finder algorithm. The models used were as follows: LG + R6 for AbiE and PDC-S05; LG + F + R6 for SoFic; LG + F + R9 for CBASS; and PDC-S27; LG + F + R8 for pAgo; LG + R + R10 for PT; LG + R10 for viperin; LG + F + R7 for PDC-M05 (subunit M05A); LG + F + R10 for PDC-S01; LG + R7 for PDC-S09; and LG + R9 PDC-S70. Ultrafast boot approximations and approximate likelihood ratio tests with one thousand replicates each to assess branch support. Phylogenetic trees were visualized using ITOL [58]. Trees were rooted at the midpoint, except for viperin and CBASS trees, which were rooted using MoaA and OAS genes, respectively, as an outgroup, following previous analyses [19, 59]. The PDC-S27 tree was rooted using AAA-ATPases (PF00004) as the outgroup.

The amino acid sequences and phylogenetic trees used to make Fig. 5 and Supplementary Fig. 7 are deposited in Supplementary Table 10 and Supplementary Data 1.

## Results

### Database creation and evaluation of defense system identification tools

We curated a comprehensive database of prokaryotic and metagenomic genomes, including 7747 archaeal and 40 000 bacterial genomes from publicly available sources (Supplementary Table 1) [30, 31]. Bacterial genomes were randomly subsampled from a total of 394 933 genomes available in the Genome Taxonomy Database (GTDB) [30], while all archaeal genomes were included. Most genomes, 92.7% of bacterial genomes and 66.9% archaeal, exceeded >80% completeness and had <5% contamination; the remainder met GTDB’s inclusion criteria of ≥ 50% completeness and < 10% contamination [30]. The taxonomic composition of the archaeal genomes is shown in Supplementary Fig. 1. As closely related strains often encode very different defense systems [6, 17], we retained these archaeal genomes to preserve resolution in system diversity.

To characterize archaeal antiviral immunity and compare it to bacterial systems, we used Padloc [16] and DefenseFinder [17]. In addition, we benchmarked CRISPR-Cas Typer [33] for the prediction of CRISPR-Cas systems, and a homology-based approach based on the REBASE database [34] for the detection of restriction-modification systems (see Methods; Supplementary Tables 2–6).

**Figure 1.**
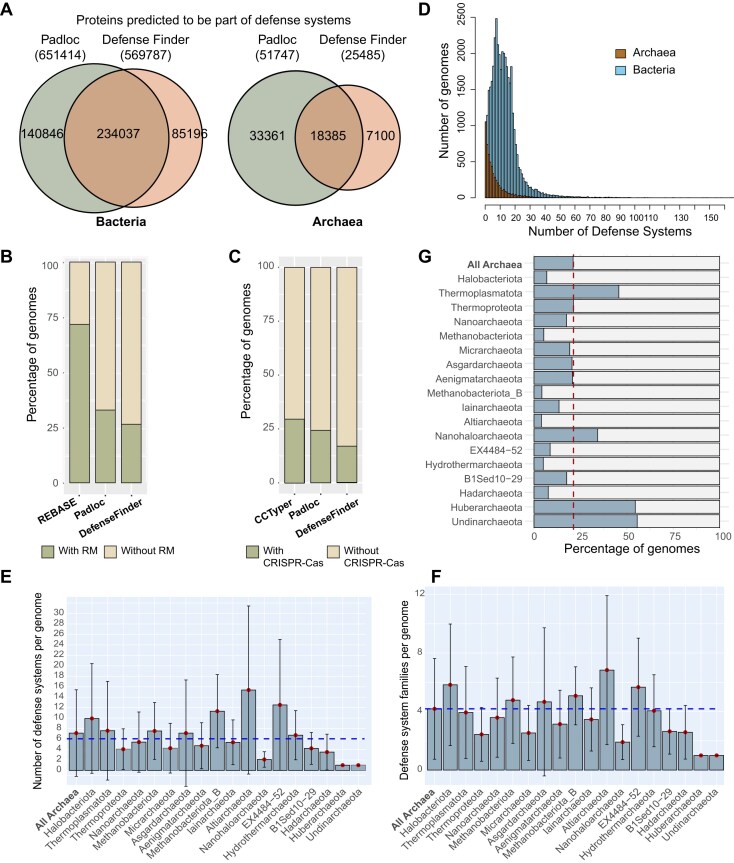
Benchmarking the identification of archaeal defense systems. (**A**) Comparison of the output from Padloc and DefenseFinder on bacterial and archaeal datasets. (**B** and **C**) Performance of tools in identifying restriction-modification systems (**B**) and CRISPR-Cas systems (**C**) within the archaeal dataset. (**D**) Distribution of defense systems per genome across prokaryotic domains. The archaeal genome with the highest number of defense systems belongs to the *Thermoplasmata* class, with 71 systems. In bacteria, members of the *Polyangeaceae* family (*Myxococcota* phylum) have the highest count, with a maximum of 167 defense systems. (**E** and **F**) Average number of defense systems (**E**) and defense system families (**F**) per genome across archaeal phyla. The dashed line indicates the average value across all Archaea. Dots represent the phylum-specific average and error bars indicate the standard deviation. (**G**) Taxonomic distribution of archaeal genomes without detected defense systems other than RM and CRISPR-Cas, shown by phylum. The dashed line indicates the domain-wide average.

To benchmark Padloc and DefenseFinder, we excluded proteins from unpublished putative candidate defense systems (PDCs) in Padloc to ensure fair comparison [15]. DefenseFinder and Padloc identified 150 and 118 systems, respectively (Supplementary Table 7). Overall, 50.9% of bacterial and 31.2% of archaeal defense proteins were identified by both tools (Fig. 1A, Supplementary Table 8). However, each tool also identified unique proteins: Padloc detected 1.6X more unique bacterial and 4.7X more unique archaeal proteins than DefenseFinder, with 56% of archaeal defense proteins uniquely detected by Padloc.

We further evaluated tool performance on RM and CRISPR-Cas system prediction. Padloc and DefenseFinder identified RM systems in 32.9% and 26.5% of archaeal genomes, respectively (Fig. 1B). These values are significantly lower than the estimated 81% prevalence reported in the REBASE database (709 archaeal genomes, October 2024) [34]. Both tools failed to detect many RM systems annotated in genomes listed in REBASE.

To improve detection, we developed a custom RM prediction pipeline based on REBASE. Archaeal proteins were matched to REBASE entries using MMseqs2 (≥65% identity, ≥80% coverage), and functional assignments were based on best hits. We further excluded proteins assigned to other defense systems by Padloc and DefenseFinder and classified RM system types based on gene neighborhood analysis (see Methods; Supplementary Table 6). This approach detected RM systems in 72.8% of archaeal genomes (Fig. 1B), consistent with previous estimates from smaller archaeal genome datasets [34, 60, 61]. Due to its higher sensitivity and agreement with prior benchmarks, we used this REBASE-based approach for all downstream RM analyses.

We also assessed CRISPR-Cas prevalence in Archaea using Padloc, DefenseFinder, and CRISPR-Cas Typer. These tools identified CRISPR-Cas systems 24.5%, 16.9%, and 29.7% of archaeal genomes, respectively (Fig. 1C). The higher detection by CRISPR-Cas Typer was primarily due to putative II-D systems, which are known to be rare in Archaea [21, 22]. Closer inspection revealed that many of these corresponded to OMEGA systems [62], including IscB-HEARO [63] (Supplementary Fig. 2), rather than true type II-D CRISPR-Cas.

In summary, Padloc provided broader coverage (Fig. 1A) and superior detection of RM (Fig. 1B) and CRISPR-Cas (Fig. 1C) than DefenseFinder. Therefore, Padloc was selected for downstream analyses of defense system distribution.

### Domain-level overview of the prokaryotic defense landscape

We identified 521 796 occurrences of defense systems (including PDCs) in bacterial genomes and 58 594 systems in archaeal genomes, comprising 1 049 452 genes in total (Supplementary Tables 2 and 6). This domain-wide analysis revealed clear differences in the distribution and diversity of defense systems between Archaea and Bacteria (Fig. 1D).

Defense system abundance deviated from a Gaussian “normal” distribution in both domains, but archaeal genomes showed a strong skew toward lower system counts per genome (Fig. 1D). Overall, 98.8% of bacterial genomes encoded at least one defense system, compared to 87.2% of archaeal genomes. Excluding RM and CRISPR-Cas, these proportions dropped to 97.84% and 68.37%, respectively (Fig. 1G), highlighting a more pronounced absence of known non-RM/CRISPR systems in Archaea.

In total, we detected 269 distinct system types in Bacteria and 197 in Archaea, with archaeal types representing 73.2% of all systems identified. Among these, only one system type, the viperin system associated with tetratricopeptide repeat-domain containing protein, was found exclusively in Archaea, consistent with previous reports [64]. While CRISPR-Cas types III-G and IV-C were only detected with archaeal genomes in our dataset, they are not considered archaeal-specific, and their functional roles as defense systems remain to be experimentally validated. By contrast, 75 defense systems were exclusive to Bacteria, comprising 16 450 occurrences (3.15% of all bacterial hits), underscoring their relative rarity.

Bacterial genomes had an average of 14.4 defense system occurrences and 5.6 distinct types per genome, compared to 6.1 occurrences and 4.2 types in archaeal genomes (Fig. 1E and F; Supplementary Fig. 3B). Known defense systems were absent across a broad range of archaeal lineages (Fig. 1G), while their absence in Bacteria was rare (1.2%) and largely limited to taxa with reduced genomes and intracellular lifestyles (Supplementary Fig. 3A), as reported previously[17].

The average genome size was 3.8 Mb for bacteria and 1.8 Mb for archaea, and a substantial fraction of archaeal genomes belong to DPANN lineages (acronym for *Diapherotrites, Parvarchaeota, Aenigmarchaeota, Nanohaloarchaeota* and *Nanoarchaeota*), organisms with small genomes and cells [65] (Supplementary Fig. 1). This raised the question whether the lower defense content in Archaea is primarily consequence of their smaller genome sizes. Genome size was moderately correlated with defense system abundance in Bacteria (Spearman ρ = 0.476, *P-*value < 2.2 × 10^−6^) and weakly correlated in Archaea (Spearman ρ=0.388, *P*-value = 1.169 × 10^−8^) (Supplementary Fig. 4A). Linear regression indicated that genome size explained 21.8% of variance in system counts per genome (R²=0.218, F[147 745]=13 330, *P *< 0.001). After accounting for genome size, residual system abundance remained significantly higher in Bacteria than in Archaea (Wilcoxon W = 1.47, *P *< 0.001), indicating that domain contributes additional variation (Supplementary Fig. 4B). Variance partitioning attributed 20% of the variation to genome size and 2% to domain, with 78.3% unexplained (Supplementary Fig. 4C). Normalizing counts per megabase confirmed that system density remained higher in Bacteria (Supplementary Fig. 4D). There results show that genome size partially explains the lower defense content in Archaea, but most variation is not attributable to genome size or domain alone, suggesting additional lineage-specific or ecological influences.

### Defense system landscapes differ between Archaea and Bacteria

**Figure 2.**
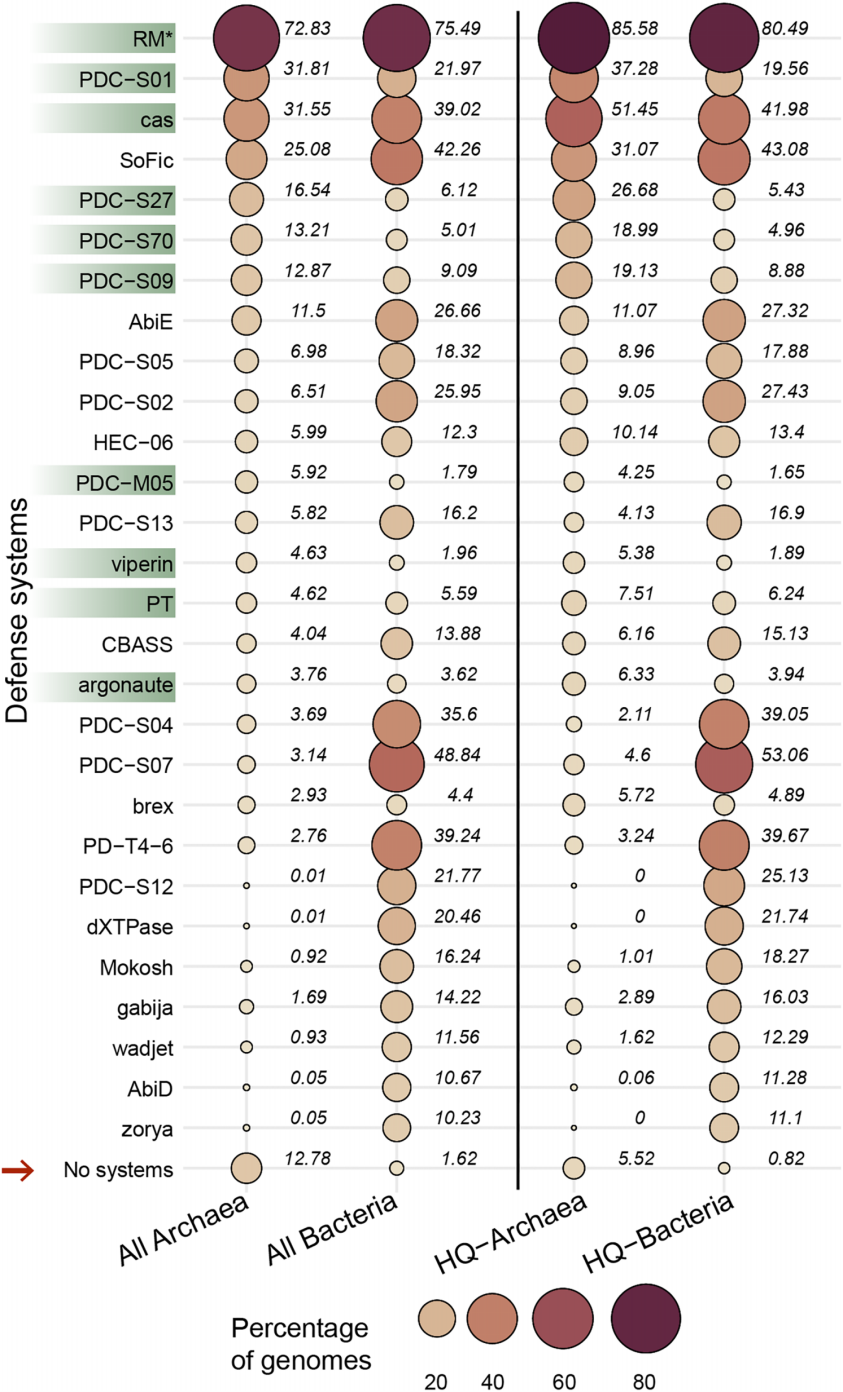
Prevalence of the 20 most abundant defense systems in Archaea and Bacteria. The percentage of genomes encoding each defense system is shown in the full datasets (left) and for high-quality (HQ) genomes only (≥90% completeness; right). Circle size and color scale with prevalence and numerical values indicate prevalence percentages. Defense systems highlighted are significantly more prevalent in Archaea than in bacteria in the HQ datasets, based on 95% confidence intervals. The arrow indicates the fraction of genomes with no defense systems detected. Prevalence values are based on PADLOC outputs, except for archaeal RM systems (*), where prevalence was calculated using the REBASE-based approach (Materials and Methods and Fig. 1B).

To compare the immune landscapes of Archaea and Bacteria, we identified the 20 most prevalent defense systems in each domain. This analysis revealed 28 systems in total, representing the most widespread components of the prokaryotic immune repertoire, hereafter referred to as the core defensome (Fig. 2).

Restriction-modification (RM) systems were the most abundant in both domains, found in 71.76% in archaeal and 75.54% bacterial genomes (Fig. 2, left panel). Despite similar prevalence, RM systems constitute a disproportionately large fraction of the archaeal defense repertoire: 38.3% of all archaeal defense proteins compared to 13.8% in Bacteria (Supplementary Fig. 5). This is not explained by higher RM copy number (2.0 vs 2.9 per genome) but likely reflects the reduced representation of other systems in Archaea, increasing RM’s relative contribution.

CRISPR-Cas systems were present in 31.1% of archaeal genomes and 39% of bacterial genomes (Fig. 2, left panel), contrasting sharply with earlier reports of 75–85% prevalence in Archaea [21, 22]. Because archaeal genomes in our dataset had lower average completeness than bacterial ones (85.2% vs 95.6%), we examined whether completeness influences measured defense prevalences. When only genomes with ≥ 90% completeness (3472 archaeal and 34 273 bacterial) were included in the analysis, archaeal prevalence increased most notably for CRISPR-Cas and SoFic, reaching 51.5% and 31.1%, respectively, whereas other archaeal systems were only mildly affected (Fig. 2, right panel and Supplementary Fig. 6). Bacterial prevalences showed comparatively minor shifts.

We further quantified the dependence of prevalence on completeness and domain using logistic regression. From this model, we estimate that CRISPR-Cas prevalence in a dataset with 100% complete genomes would be ∼55% (95% CI: 53.1–56.7%) in Archaea and ∼43% (95% CI: 42.7–43.8%) in Bacteria (Supplementary Fig. 6). Thus, incomplete genomes account for a substantial part, but not all, of the discrepancy with earlier reports [66], and our estimates are consistent with a recent estimate of 52% prevalence of CRISPR-Cas in Archaea [67], published while this work was under review.

Interestingly, six of the ten most prevalent systems are putative defense candidates (PDCs) (Fig. 2), single-gene systems recently identified through a “guilt-by-embedding” approach [15]. Several Hma-embedded candidates (HECs), including HEC-06, have demonstrated antiviral activity in experimental assays[15], but experimental validation is pending for most PDCs in the core defensome. PDC-S01 is the fourth most common defense across all prokaryotes, and five PDCs (PDC-S01, PDC-S27, PDC-S70, PDC-S09, and PDC-M05) are more abundant in Archaea than in Bacteria, highlighting the relevance of PDCs to archaeal immunity and their potential as targets for future characterization.

Among the 15 experimentally validated antiviral defense systems in the core defensome, only viperins are more prevalent in Archaea than in Bacteria, and argonautes and PT showed increased prevalence in Archaea after adjusting for completeness (Fig. 2 and Supplementary Fig. 6). All other validated antiviral systems are more common in Bacteria, suggesting either greater evolutionary diversification in this domain, differences in cellular machineries across domains that may limit system compatibility or operation, or archaeal underrepresentation in current defense models.

### Lineage-dependent differences in the archaeal immune pangenome

**Figure 3.**
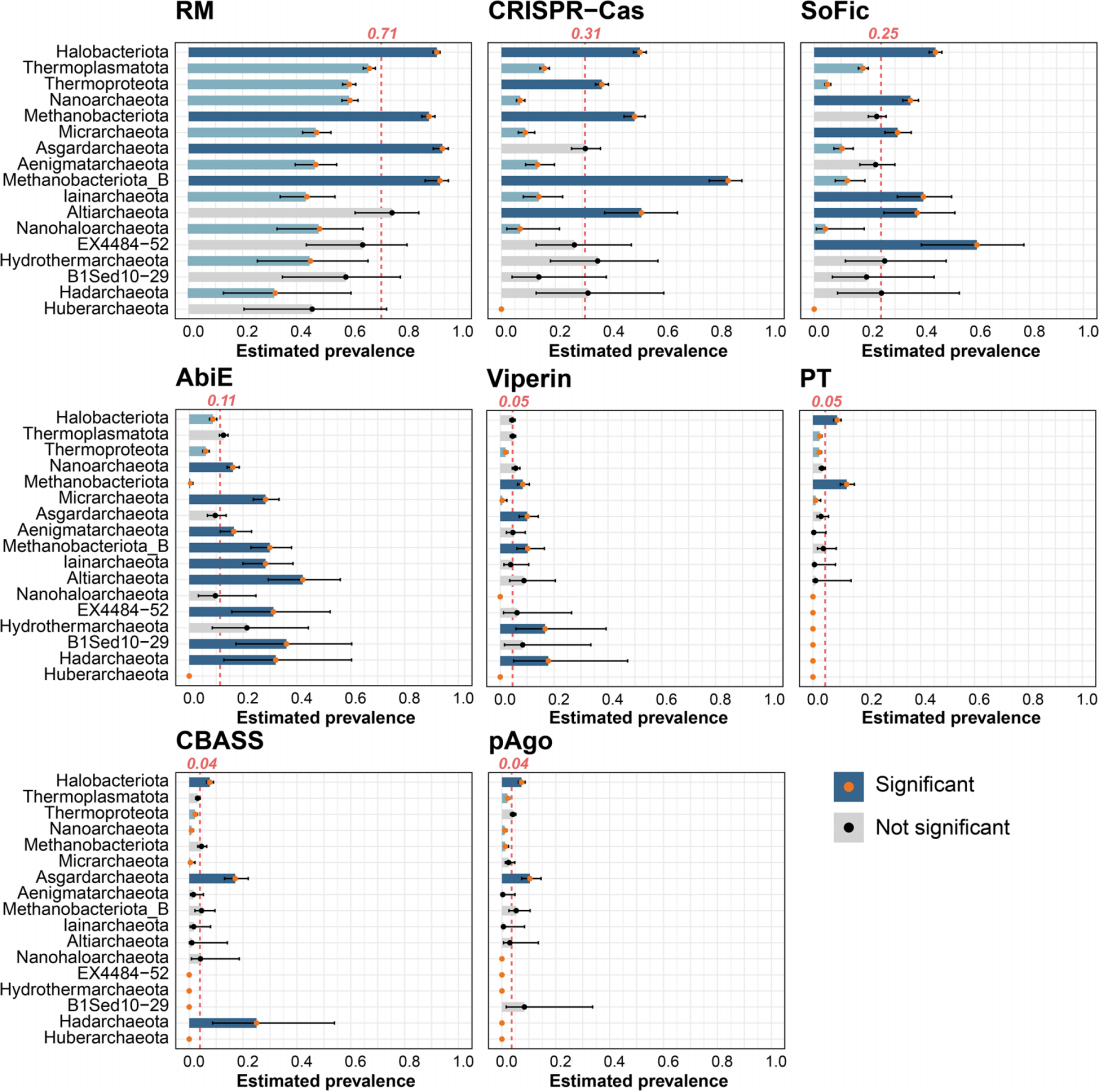
Taxonomic distribution of the archaeal core-defensome. The bar charts represent the percentage of genomes in each phylum containing the defense system. The red dashed line indicates the average prevalence of each defense system across all archaeal genomes. Blue bars indicate a significant difference in the prevalence of a system against the overall archaeal mean (dark blue, overrepresented; light blue, underrepresented). Error bars indicate the confidence intervals.

We analyzed the prevalence of experimentally validated antiviral systems with >3% prevalence across archaeal phyla (*n *≥ 10), namely RM, CRISPR-Cas, SoFic, AbiE, viperin, DNA phosphorothioation, CBASS, and argonaute systems using binomial GLM models fitted to presence/absence data and estimated marginal means to compare each phylum against the overall archaeal mean (Fig. 3 and Supplementary Table 9). These systems show heterogeneous (“patchy”) distribution across archaeal lineages, a hallmark of defense system evolution likely shaped by frequent horizontal gene transfer [17]. Similar patterns are observed for PDCs (Supplementary Fig. 7).

RM and CRISPR-Cas are underrepresented in DPANN archaea, which instead show enrichment in AbiE, SoFic, and multiple PDCs. This mirrors patterns seen in host-dependent bacteria with small genomes (e.g. *Chlamydiota* and *Patescibacteria)*, suggesting a selective pressure for compact, single-gene systems in symbiotic or parasitic lineages (Supplementary Figs 3 and 7).

*Halobacteriota* show broad enrichment for both core and PDC defenses (Fig. 3, Supplementary Fig. 7), with few underrepresented exceptions (e.g. Mokosh, PDC-S07). In contrast, *Thermoproteota* generally show lower defense prevalence, except for RM, CRISPR-Cas, and argonautes. *Thermoplasmatota* exhibit low prevalence of CRISPR-Cas, but retain RM, AbiE, viperin, CBASS, and several PDCs (e.g. PDC-S01, PDC-S27, and PDC-S04). *Asgardarchaeota* genomes are enriched in RM, viperins, CBASS, argonautes, and selected PDCs (S70, S09), but show underrepresentation of SoFic and most other PDCs (Fig. 3, Supplementary Fig. 7).

**Figure 4.**
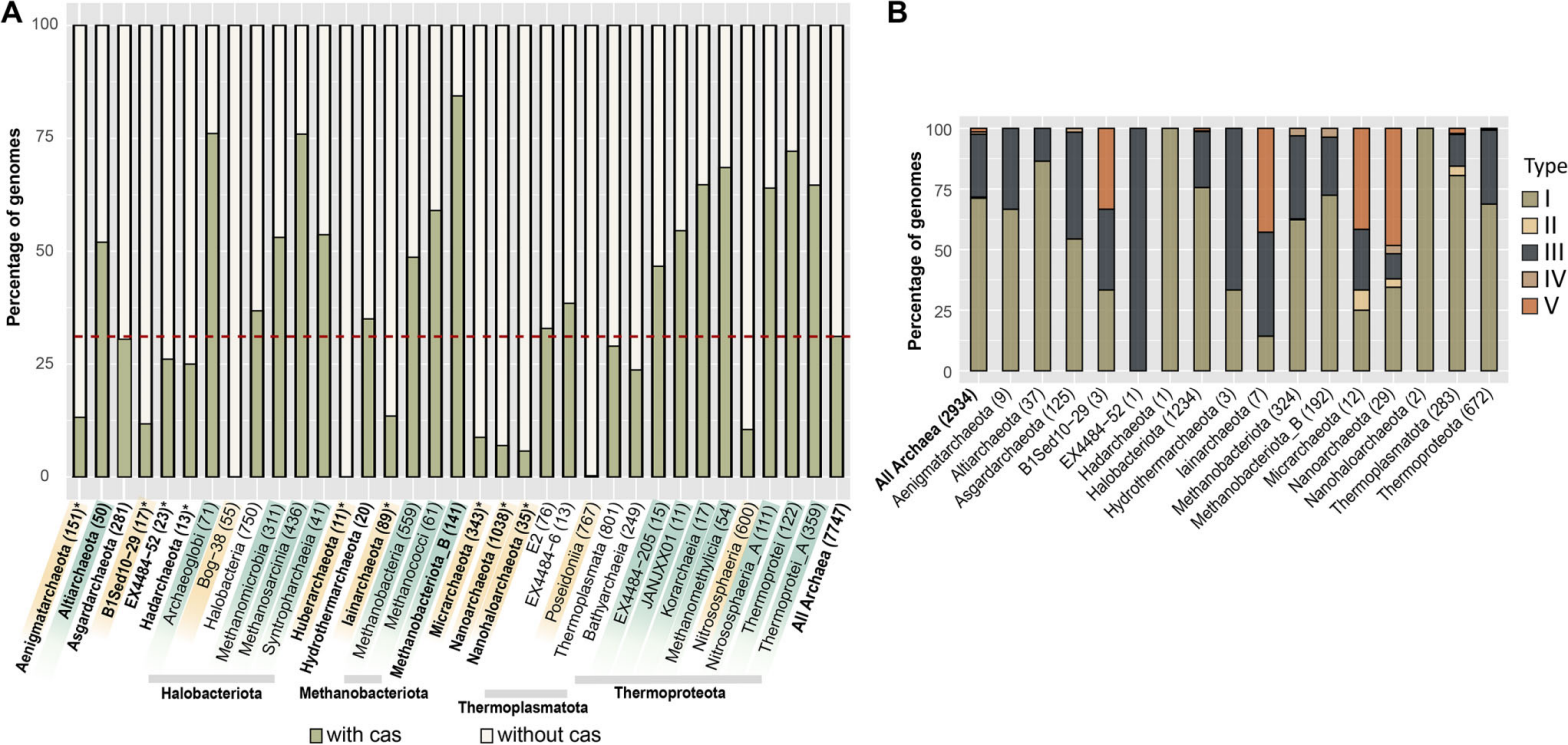
CRISPR-Cas distribution in Archaea. (**A**) Prevalence of the CRISPR-Cas system across archaeal lineages, with the red line indicating the average prevalence for the domain. Phylum names are in bold, while class-levels are shown in regular font. Lineages highlighted in green have above-average CRISPR-Cas prevalence, while those in yellow have below-average prevalence. Asterisk (*) denotes DPANN lineages. (**B**) Relative abundance of CRISPR-Cas types across archaeal phyla. Numbers in parentheses indicate the total genomes analyzed for each lineage.

CRISPR-Cas prevalence also varied across temperature classes. It was the highest in the hyperthermophilic *Methanobacteriota_B* phylum and in thermophilic classes of the *Thermoproteota* and *Halobacteriota* (>50% prevalence) compared to their mesophilic counterparts (10–25% and 0–37%, respectively), and was also enriched in the mesophilic *Altiarchaeota* (∼50%) (Fig. 4A). In contrast, CRISPR-Cas are notably underrepresented in DPANN archaea, across all temperature categories (Fig. 4A). The distribution of CRISPR-Cas types remains consistent with prior studies: type I and III dominate, while type II and IV are rare, and type VI is absent (Fig. 4B) [21, 22].

Because optimal growth temperature varies within lineages, we predicted optimal growth temperature (OGT) for all archaeal genomes using Tome and analyzed the relationship between OGT and core system abundance using negative binomial generalized linear models (Supplementary Fig. 8). CRISPR-Cas abundance is positively correlated with increased OGT, with a 35.7% increase in Cas counts per 10°C increment (*P *< 0.001). RM and SoFic show negative relationships with OGT (−13.2% and −42.2% per 10°C, respectively; both *P *< 0.001) (Supplementary Fig. 8). These associations explain ∼13.4–15.4% of the variance (Nagelkerke’s pseudo-R^2^). Weak but significant negative associations were identified for PT and CBASS, and a positive association for argonautes, although these account for <1% of the explainable variance (Supplementary Fig. 8). These results are consistent with previous reports linking higher CRISPR-Cas abundance to hosts with high optimal growth temperatures and SoFic enrichment in lower-temperature hosts [68].

Next, we quantified the contribution of each factor to defense abundance for each domain using Nagelkerke´s pseudo-R^2^ (Supplementary Fig. 4E). Genome size explained the largest fraction of variability in both domains, followed by phylum and completeness. Optimal temperature contributed negligibly in Archaea. Even though completeness and temperature contribute smaller effects at the whole-defensome scale, they strongly influence the prevalence of specific systems such as CRISPR-Cas and SoFic, underscoring the importance of system heterogeneity. When all predictors were combined, they explained 38.5% of the variation in Archaea and 36.6% in Bacteria (Supplementary Fig. 4E). These predictors only partially explain the variation in archaeal defense content, yet bacteria encode substantially more defenses and are similarly affected by these predictors. This suggests that other processes, such as HGT, mobile genetic elements, genome reduction, symbiosis, viral pressure, and/or methodological biases, play dominant roles in shaping archaeal immune repertoires.

### Evolutionary origins of the prokaryotic core-defensome

While the evolutionary trajectories of RM and CRISPR-Cas systems have been extensively reviewed elsewhere [20, 22, 60, 61, 69–73], we focus here on the remaining components of the prokaryotic immune repertoire.

Several innate components in eukaryotes are evolutionarily linked to prokaryotic systems [27]. Although many such systems likely originated in Bacteria, others, such as viperins and argonautes, appear to have archaeal origins, particularly within *Asgardarchaeota*, the closest relatives of eukaryotes [18]. These systems are especially prevalent i*n Asgardarchaeota* but are also found across other archaeal phyla (Fig. 3).

**Figure 5.**
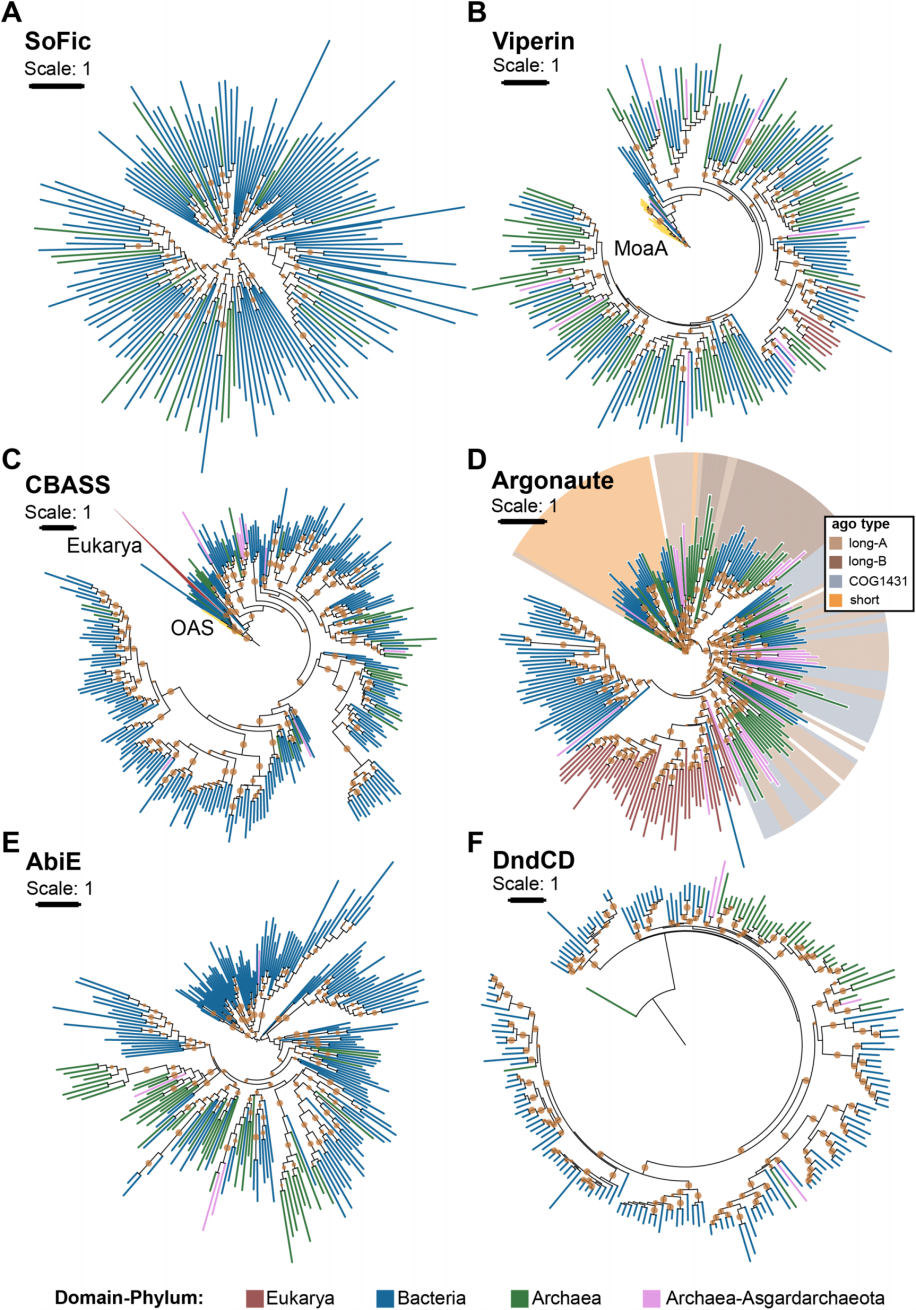
Evolutionary origins of the core-defensome. Phylogenetic trees of systems in the archaeal core-defensome. Branch colors indicate taxonomic classification: blue for bacteria, red for eukaryotes, green for archaea, and pink for Asgard archaea. The argonaute tree was constructed from candidates identified with Padloc, and colored ranges indicate DefenseFinder subtype HMM-profile annotations (long-A, long-B, short, or COG1431_pAgo) when available; leaves without colored ranges correspond to eukaryotic eAgos or Padloc-predicted argonautes not classified by DefenseFinder. Bootstrap support values (≥70%) are marked as dots at the corresponding nodes. Trees for viperins (**B**) and CBASS (**C**) were rooted using MoaA and 2′-5′-oligoadenylate synthetase (OAS) sequences, respectively, while other trees were midpoint-rooted (**A, D–F**).

To explore whether the current distribution of core defense systems reflects vertical inheritance or horizontal gene transfer (HGT), we analyzed the phylogenies of archaeal and bacterial homologs. In agreement with previous studies [51, 59, 64, 74], our trees support archaeal viperins and argonautes as ancestral immune systems, with deep phylogenetic roots (Fig. 5B

and D). Both systems show patchy distribution with multiple inter-domain HGT events, but they are notably overrepresented in *Asgardarchaeota*, which make up only 3.6% of the archaeal dataset but account for 7.4% of archaeal viperins and 16% of archaeal argonautes (Fig. 3).

Homologs of both systems are also found in eukaryotes [51, 64], and our analysis supports an archaeal origin for eukaryotic argonautes (eAgo) and specifically from long-A argonautes, consistent with previous work [51, 74, 75]. Most asgardarchaeal argonautes fall within the clade from which eAgos appear to have originated (Fig. 5D), agreeing with previous work [18]. Similarly, the largest eukaryotic viperin cluster forms a sister group to a clade of archaeal proteins, including most asgardarchaeal viperins (Fig. 5B and Supplementary Fig. 9). While this supports a likely archaeal origin for eukaryotic viperins, our analysis does not pinpoint a specific contributing phylum.

Our results also align with those of Culbertson and Levin [59], who identified several bacterial-to-eukaryote HGT events involving viperins. However, in contrast to Shomar *et al.* [19], who reported clear phylogenetic separation between archaeal and bacterial viperins, we observed no strict domain-based division (Fig. 5B and Supplementary Fig. 9).

In contrast, systems such as SoFic, CBASS, AbiE, and the DNA phosphorothioation (Dnd) appear to have a likely bacterial origin, with phylogenies indicating multiple bacteria-to-archaea transfer events (except for PT, displaying a high degree of domain-separation) (Fig. 5 A, C–E). While our phylogenies suggest likely origins for several systems, alternative scenarios, such as vertical inheritance and differential loss, cannot be excluded, even if they are less supported by the current data. Notably, while archaeal AbiE systems share a bacterial ancestor, they tend to cluster separately, suggesting some degree of domain-specificity (Fig. 5D). AbiE is a type IV toxin-antitoxin system known to induce abortive infection by acting on an unknown cellular target [76, 77], and its apparent domain restriction may reflect differences in host-specific targets.

PDCs display diverse evolutionary histories. Systems such as S09, S05, S02, S13, S04, S07, S12 and HEC-06 are predominantly found in bacteria and were likely acquired by Archaea via HGT and show limited subsequent diversification (Supplementary Fig. 10). Others, like S01, S27, S70 and M05 are notably enriched in Archaea, suggesting a possible archaeal origin for these systems (Supplementary Fig. 10).

Among these, PDC-S01 and PDC-S27 are especially abundant in Archaea, accounting for 25% and 43% of their total detected occurrences, respectively. In absolute numbers, we detected 9986 S01 and 3503 S27 systems in Archaea versus 29 494 and 4562 in Bacteria, respectively. PDC-S01 has undergone multiple transfers across domains, followed by some degree of domain-specific diversification (Supplementary Fig. 10). Conversely, S27 appears to have originated in Bacteria and spread into Archaea (Supplementary Fig. 10). Structurally, both encode ATPase domains fused to members of the PD-(D/E)-X-K superfamily, associated with nucleic acid targeting [15].

PDC-M05 is even more archaeal-enriched (59% of all detected instances) (Supplementary Fig. 10). Its architecture, composed of a nucleotidyltransferase and a HEPN-domain protein, is characteristic of known prokaryotic toxin-antitoxin modules [78] [79], although its antiviral role remains to be confirmed. Similarly, PDC-S70, which encodes a PIN nuclease domain, is predominantly archaeal (56% of all counts) and shows deep phylogenetic signal consistent with a possible archaeal origin (Supplementary Fig. 10), followed by multiple subsequent transfers into Bacteria.

Although most PDCs lack experimental validation, their high representation in Archaea and molecular features suggest they are functionally relevant and may represent archaeal contributions to the immune repertoire. These systems are strong candidates for future characterization.

## Discussion

Archaea remain the least studied domain of life, including their antiviral strategies. While CRISPR-Cas immunity has been characterized in detail [20, 80], the broader archaeal immune landscape has received little attention, limiting our understanding of the evolution of innate immunity across life.

We present the most taxonomically comprehensive analysis of archaeal antiviral defenses to date and show that Archaea encode fewer and less diverse known defense systems than bacteria, even after accounting for genome completeness and lineage structures. Revised prevalence estimates for CRISPR-Cas (∼50–55%) using this archaeal-enriched dataset indicate that earlier figures based on limited sampling overestimated archaeal CRISPR prevalence and highlight the impact of archaeal underrepresentation and methodological biases in current defense detection frameworks.

A major challenge in benchmarking archaeal immunity lies in the available detection frameworks. Tools such as DefenseFinder and Padloc rely primarily on homology-based searches to identify defense genes. These approaches have been developed using large bacterial datasets, bacterial-centric protein profiles, and antiviral activity validated in models such as *E. coli* [11–13], and their sensitivity and specificity for archaeal homologs remain to be evaluated. Importantly, domain sharing among defense systems could potentially lead to misclassification of unrelated proteins as defense systems, an issue particularly relevant for single-gene systems. However, these tools are currently the most systematic approach for prokaryotic defense system detection and are widely adopted in the field.

Archaeal genomes are also underrepresented in genomic databases (only ∼2% of high-quality assemblies [30]), frequently incomplete, and typically originate from uncultivated or genetically intractable lineages [23, 81–83]]. Detection of archaeal homologs of various bacterial and eukaryotic proteins, including, but not limited to, antiviral systems, often requires custom approaches such as novel HMM profiles or structure-guided searches [84–90]. Given archaeal-specific features in information processing and cell envelope biology, some antiviral strategies may be mechanistically distinct enough to escape homology-based detection or favor the evolution of new defense systems, further contributing to the apparent disparity between archaeal and bacterial immune landscapes. Therefore, observed archaeal prevalence values should be interpreted conservatively, and comparisons to bacterial systems must account for these detection biases.

Explaining this archaeal-bacterial disparity in defense content remains challenging because genome size, phylum, genome completeness, or temperature only partially account for the observed variation. Ecological factors, particularly viral dynamics, may offer a more compelling explanation. Previous work has shown that environments with high viral abundance and low viral diversity are associated with higher CRISPR-Cas prevalence, whereas phylogenetic relatedness plays a limited role [91]. Symbiotic or parasitic lifestyles may further influence the selection of specific defense systems. However, they are unlikely to fully account for the general bias towards reduced defense content and diversity observed across archaeal lineages.

Despite these limitations, our analysis reveals distinct features of archaeal immunity. Many of the most-prevalent defense systems are single-gene putative defense candidates (PDCs), many of which are not associated with known defense islands [15]. Although PDCs remain unvalidated, their abundance and taxonomic breadth point to a large pool of overlooked systems. Interestingly, three prevalent PDCs (S01, S70, and M05) are ancestral and likely of archaeal origin, rare examples (together with CRISPR-Cas) of putative archaeal-derived contributions to the prokaryotic immune repertoire.

These findings highlight the need for archaeal-adapted discovery strategies. Recent approaches such as regulatory motif mining in archaeal viruses [92], machine-learning [93], and guilt-by-embedding analysis [15] have already uncovered hundreds of new candidate anti-defense genes and defense systems. Moreover, exploring genomic regions enriched in mobile genetic elements (MGEs), beyond classical defense islands, has proven fruitful in both Bacteria [94–98] and Archaea [99, 100], and will be essential for capturing the full diversity of archaeal immunity. The discovery of viral inhibitors of archaeal defenses remains limited [101–105], further underscoring the need to expand beyond conventional approaches.

Our evolutionary analyses align with previous observations that defense systems evolve primarily through horizontal gene transfer rather than vertical inheritance and reinforce the view that eukaryotic immunity is a mosaic shaped predominantly by bacterial, and to a lesser extent archaeal, contributions. Eukaryotic homologs of viperins and argonautes appear to have originated in Archaea, highlighting the evolutionary relevance of archaeal immunity. While alternative evolutionary scenarios may exist, they are not strongly supported by the current data. Finally, Archaea’s lower global biomass (∼10% of bacterial biomass) [106] may limit both the genomic diversity and the frequency of inter-domain encounters, contributing to the observed bias toward bacteria-to-archaea [107] (and bacteria-to-eukaryote) horizontal transfers, where the latter are thought to underlie the bacterial origins of several innate immune systems in eukaryotes [27, 59].

In conclusion, within the limits of currently recognized defense families and annotation tools, Archaea appear to encode fewer and less diverse known defense systems than Bacteria. Predictors such as genome size, optimal growth temperature, assembly completeness, and taxonomy grouping explain part of the within-Archaea variation, but whether cross-domain disparity reflects true ecological differences, methodological underdetection, or the presence of archaeal-specific strategies remains unclear and will be the focus of future research. Our results challenge the assumption that archaeal and bacterial immune landscapes are broadly similar and emphasize the need for archaeal-adapted tools and validation strategies to uncover the full diversity of archaeal immunity and, by extension, deepen our understanding of immunity across all domains of life.

## Supplementary Material

gkag225_Supplemental_Files

## Data Availability

Scripts are available at: https://github.com/laura-MtA/Martinez-Alvarez_et.al._2026 and Zenodo at https://doi.org/10.5281/zenodo.18757827.
