# Supplementary material for: Diversity and evolution of archaeal immune strategies
Source: Nucleic Acids Res. 2026 Mar 14;54(5):gkag225. doi: 10.1093/nar/gkag225 (PMC12988326; doi:10.1093/nar/gkag225)
Supplement: gkag225_Supplemental_Files [file gkag225_supplemental_files.zip › Martinez-Alvarez25.NAR.SupplementaryMaterial.Revised.Unmarked.docx]

**Diversity and evolution of archaeal immune strategies**

Laura Martínez-Alvarez^1^*, Xu Peng^1^*

*To whom correspondence should be addressed. Email: [laura.martinez@bio.ku.dk](mailto:laura.martinez@bio.ku.dk), [peng@bio.ku.dk](mailto:peng@bio.ku.dk)

**This file includes:**

Supplementary Figures 1 to 10

**Other Supplementary Files for this manuscript include:**

Supplementary Tables 1 to 10 and Supplementary Data 1.

**SUPPLEMENTARY FIGURES**


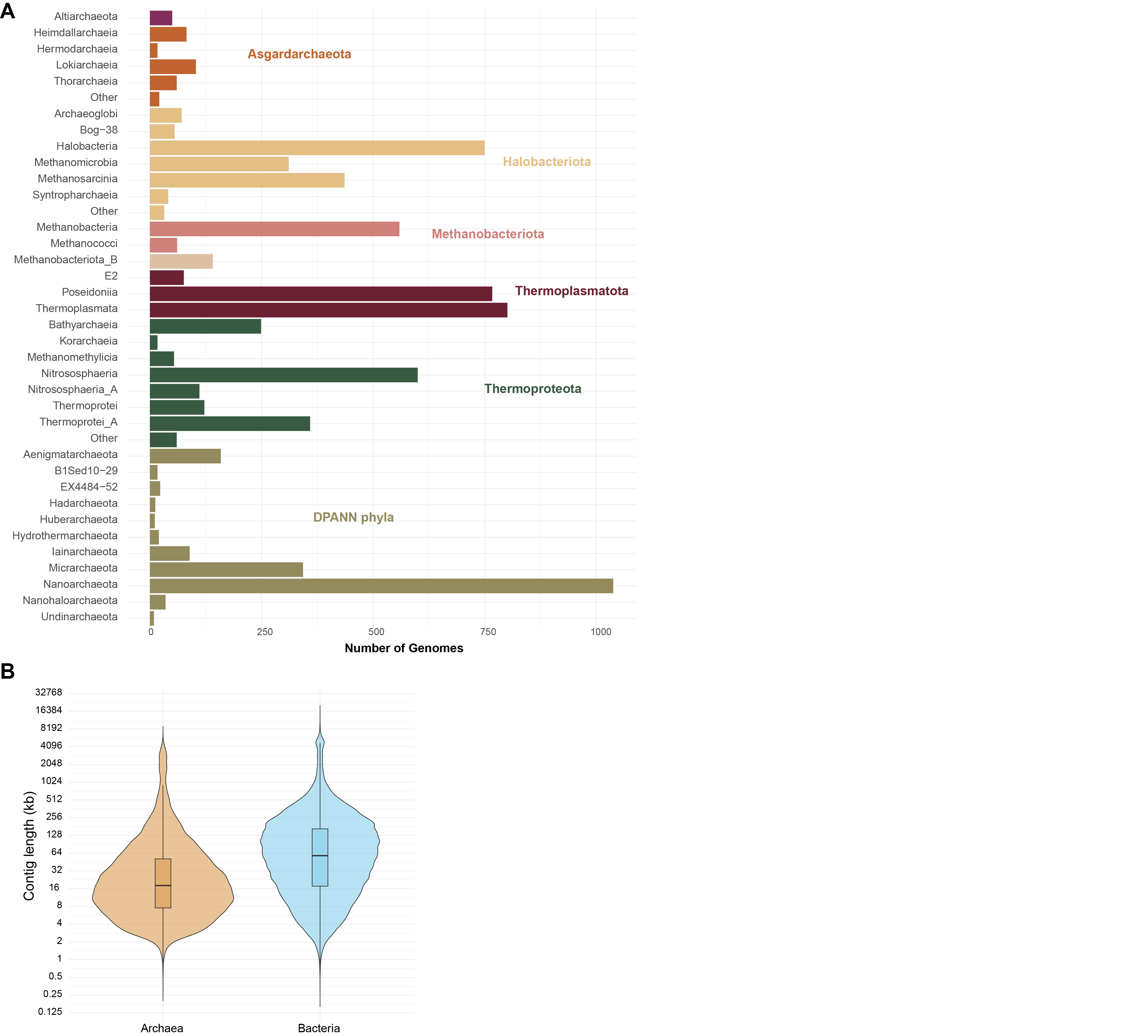

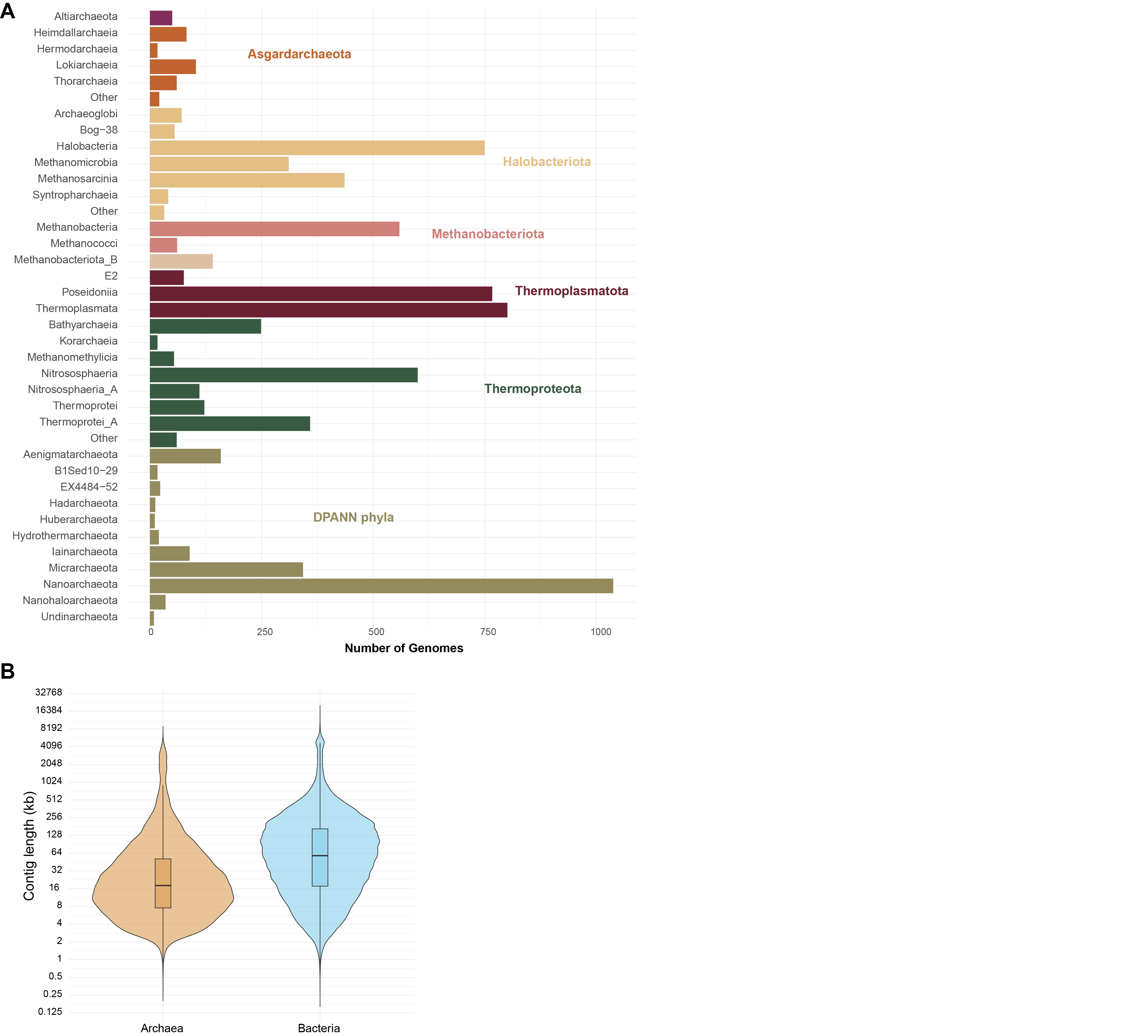


**Supplementary Figure 1. Taxonomic diversity of the archaeal database. A)** The taxonomic classification of genomes follows the phylogeny of the Genome Taxonomy Database (Rinke et al. 2021). Bars are colored according to the phylum or superphylum to which they belong. B) Contig-length distribution of defense system-encoding sequences. Violin and boxplots showing the distribution of contig lengths (in kilobases). The y-axis is log_2_-scaled to improve visualization. Boxplots indicate median and interquartile ranges; violins represent kernel density estimates.

**Supplementary Figure 2. Phylogeny of archaeal type-II CRISPR-Cas systems identified by CRISPR-Cas Typer.** Proteins from the archaeal database are indicated in black and representative sequences of the previously characterized Cas9 subtypes (II-A to II-D) or IscB elements are shown in color. Sequences belonging to OMEGA clades (Altae-Tran et al. 2021, Aliaga**
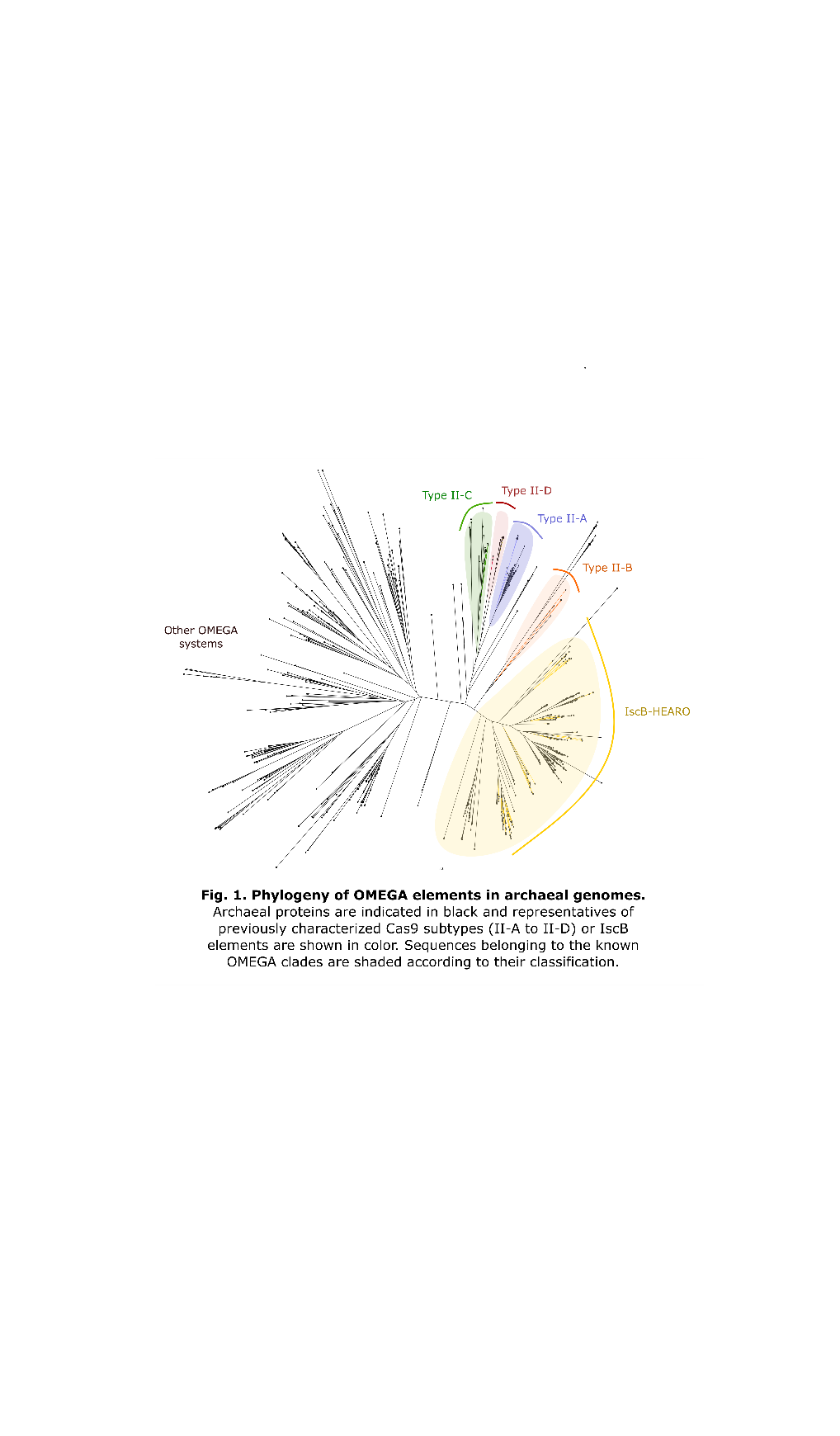
**-Goltsman et al. 2022) are shaded in yellow. Proteins identified as type II-D CRISPR-Cas effectors do not cluster with the known Cas9 subtypes and are likely OMEGA systems.

**
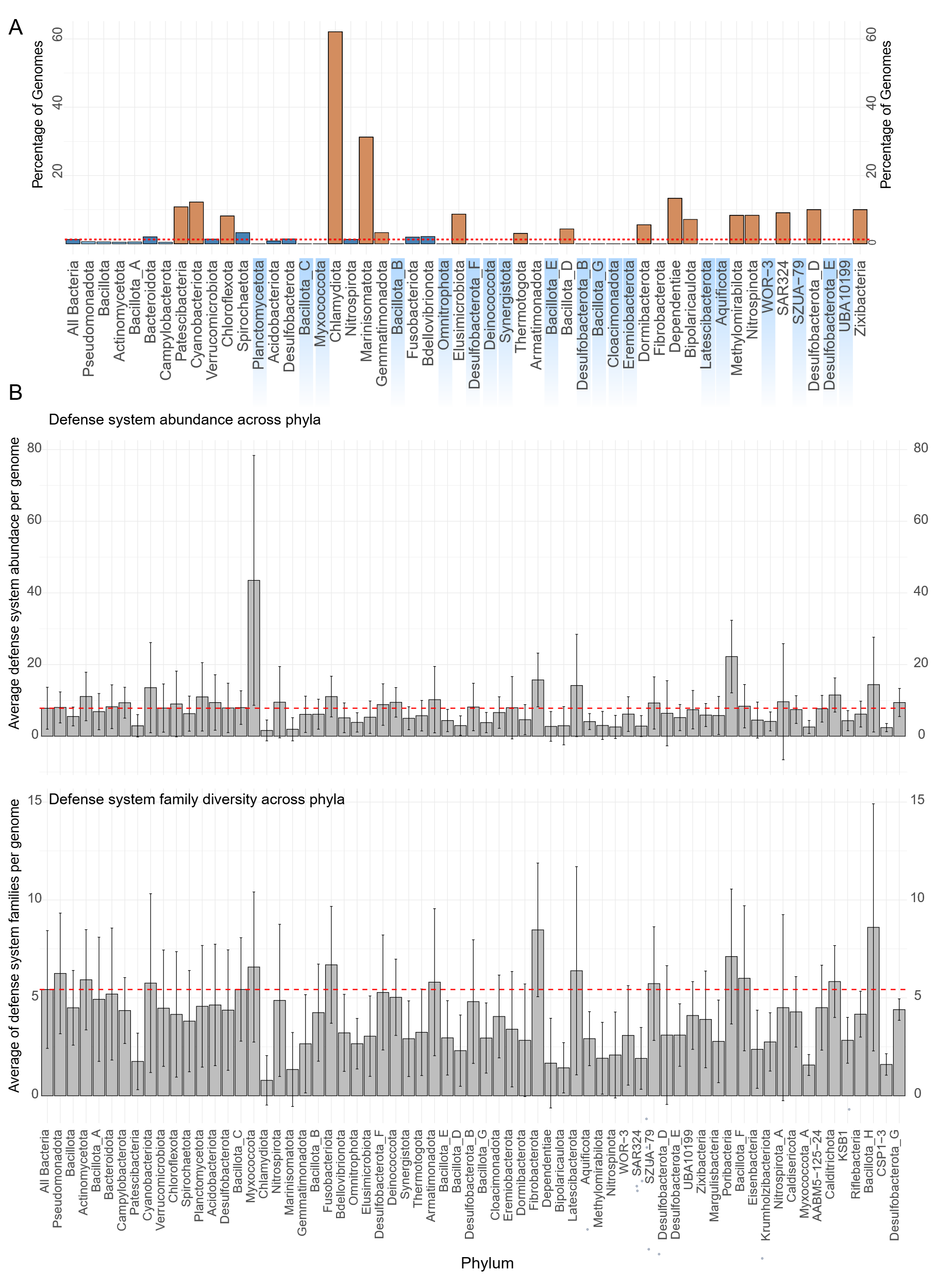
**

**Supplementary Figure 3. Statistical analysis of bacterial defense systems. A)** Taxonomic distribution of bacterial genomes lacking defense systems. The dashed red line indicates the average percentage across all bacterial genomes analyzed. Orange bars indicate above-average values. All genomes belonging to the phyla shaded in blue encode one or more defense system. **B)** Comparison of defense system abundance and diversity across bacterial phyla. The top panel shows the average number of defense systems per genome. The bottom panel presents the average number of defense system families per genome. The dashed red line indicated the average value of all bacterial genomes analyzed. Error bars indicate standard deviation.


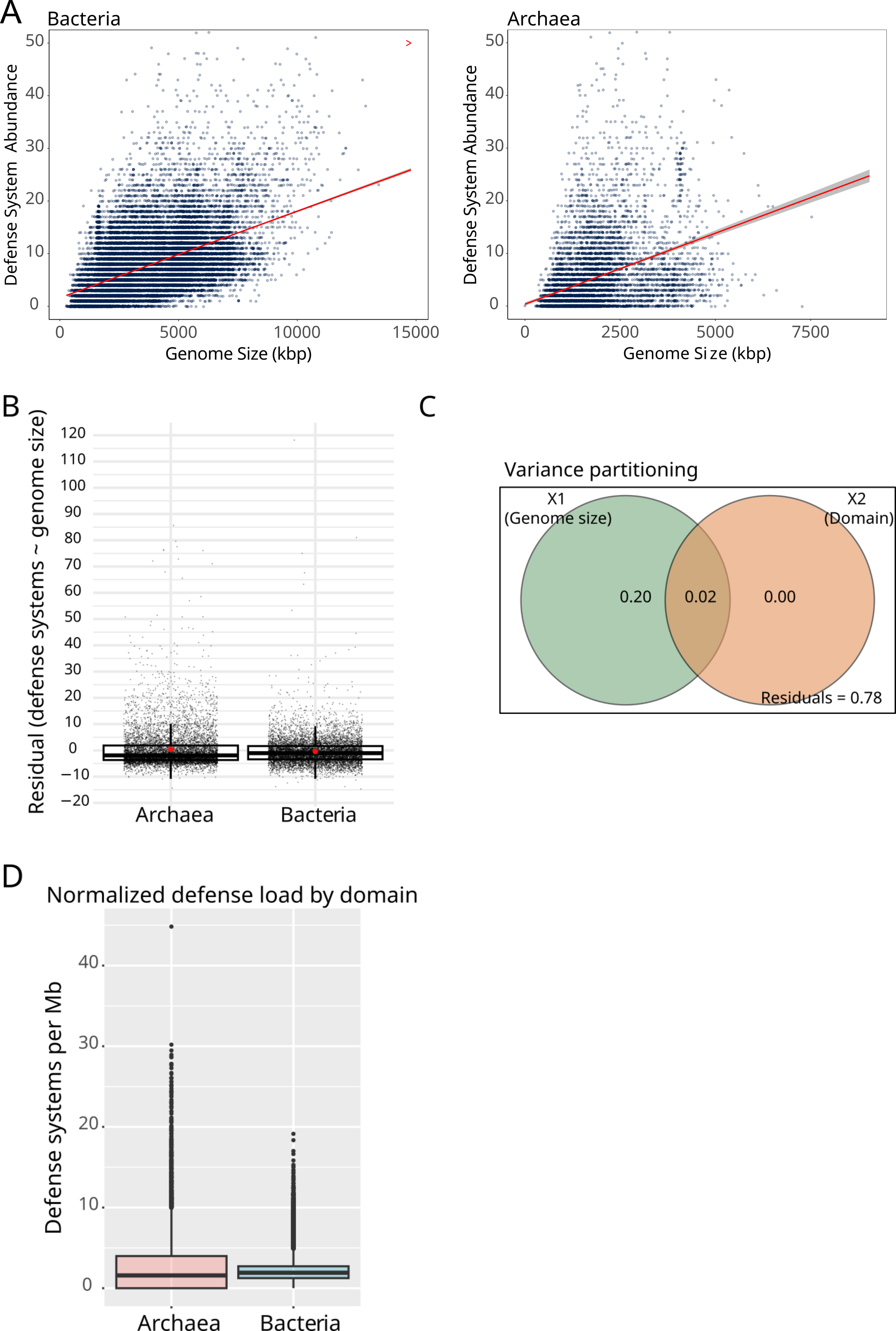


Spearman:ρ=0.476, *p-*value < 0.001

Spearman: ρ=0.388, *p* -value < 0.001

E


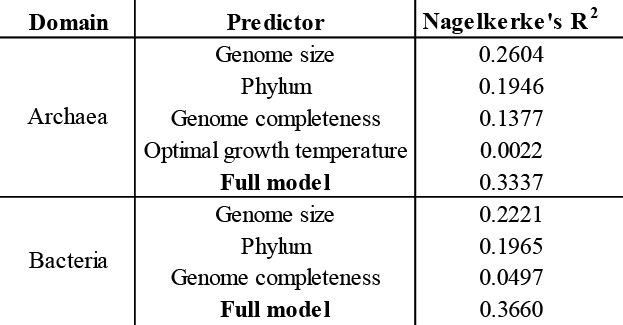


**Supplementary Figure 4. Genome size and domain explain only a small fraction of the variance in defense system content. A)** Relationship between genome size and defense system abundance in Bacteria (Left) and Archaea (Right). Each dot represents a genome, with the number of defense systems plotted against genome size. The red line represents the linear regression trendline, while the shaded area indicates the standard error of the regression fit. **B)** Residuals from a linear model predicting total defense systems by genome size, stratified by domain. Archaea have higher mean (+ 0.548) but lower median residual (-1.80) than Bacteria (- 0.106 and - 0.796, respectively), indicating greater heterogeneity and more frequent undersaturation relative to genome size. A Wilcoxon rank-sum test confirmed significant difference between domains (*W* = 1.47*10^8^, ρ < 0.001) **C)** Variance partitioning shows that genome size accounts for 20% of variance, domain for 2%, while the majority remains unexplained (78%). **D)** Defense systems normalized per megabase of genome. Bacteria exhibit a significantly higher density of defense systems per Mb compared to Archaea. **E)** Nagelkerke´s pseudo-R^2^ values for negative binomial models quantifying the contribution of individual genomic and ecological predictors to defense abundance in Archaea and Bacteria. Values correspond to one-predictor models fitted with log-transformed genome size, phylum, genome completeness or optimal growth temperature (Archaea only). Full model includes all predictors for the corresponding domain.

**
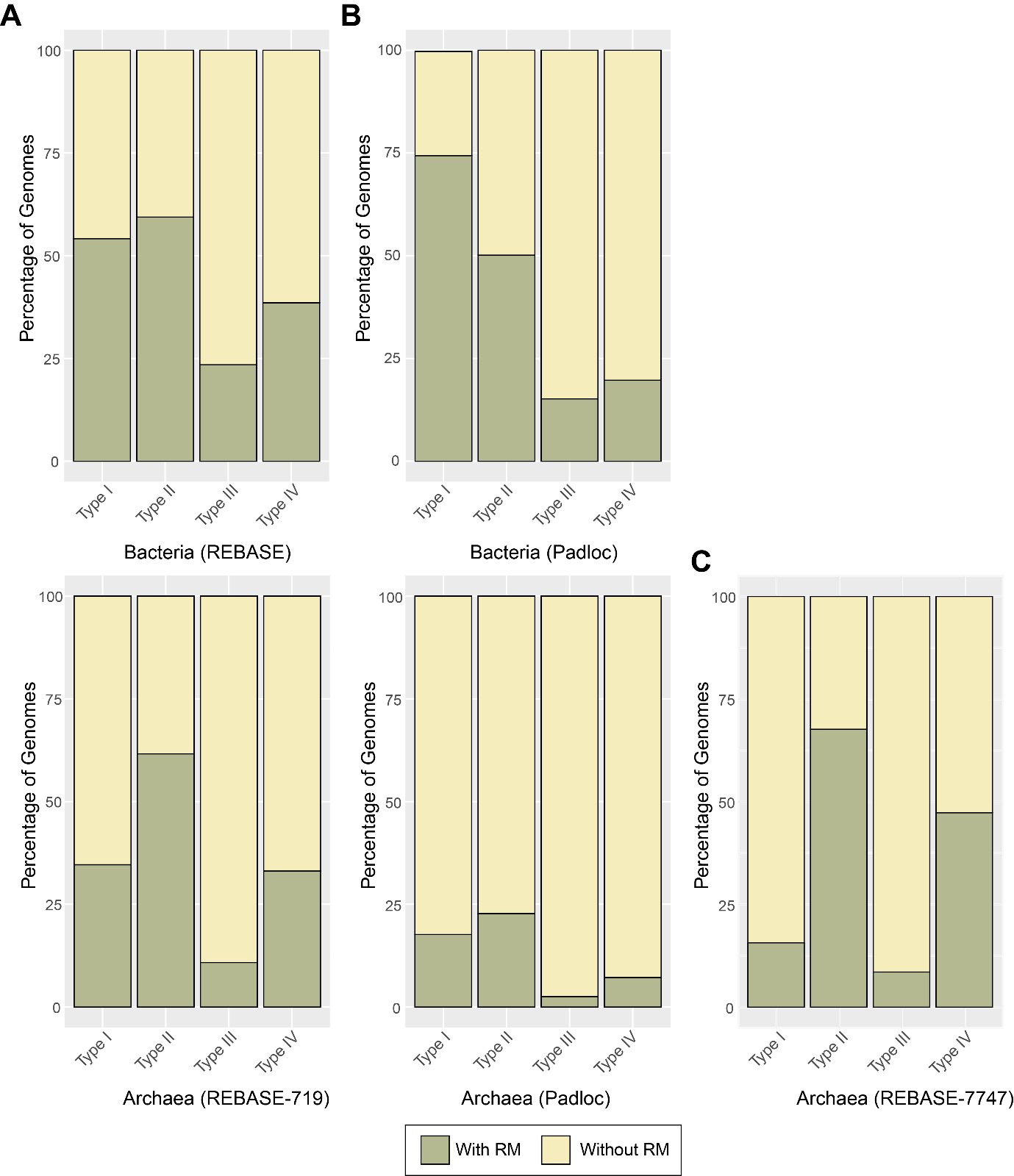
**

**Supplementary Figure 5. Output of the restriction-modification identification approaches.** Prevalence of the four types of restriction-modification systems in the genomes deposited in REBASE (Roberts et al. 2023) **(A),** or in the bacterial and archaeal datasets used in this work as predicted by PADLOC **(B)** or our homology REBASE-based approach (REBASE-7747) **(C)**.


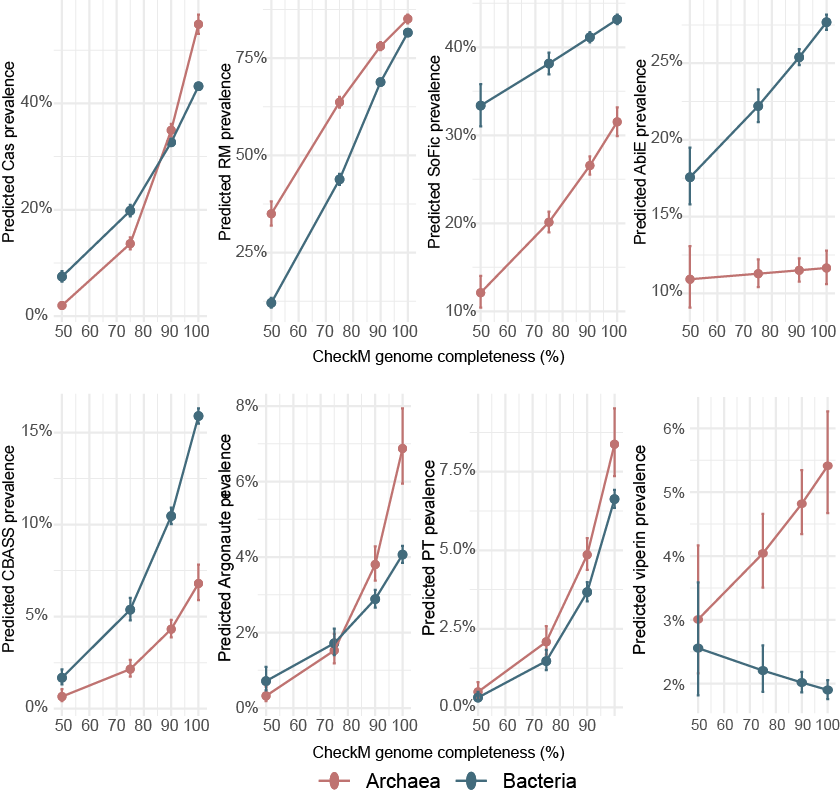


**Supplementary Figure 6. Effect of genome completeness on defense system prevalence.** Predicted prevalence of core defense systems as a function of genome completeness. Lines show predicted prevalence from logistic models for each core system. Error bars represent 95% confidence intervals (Wald method).


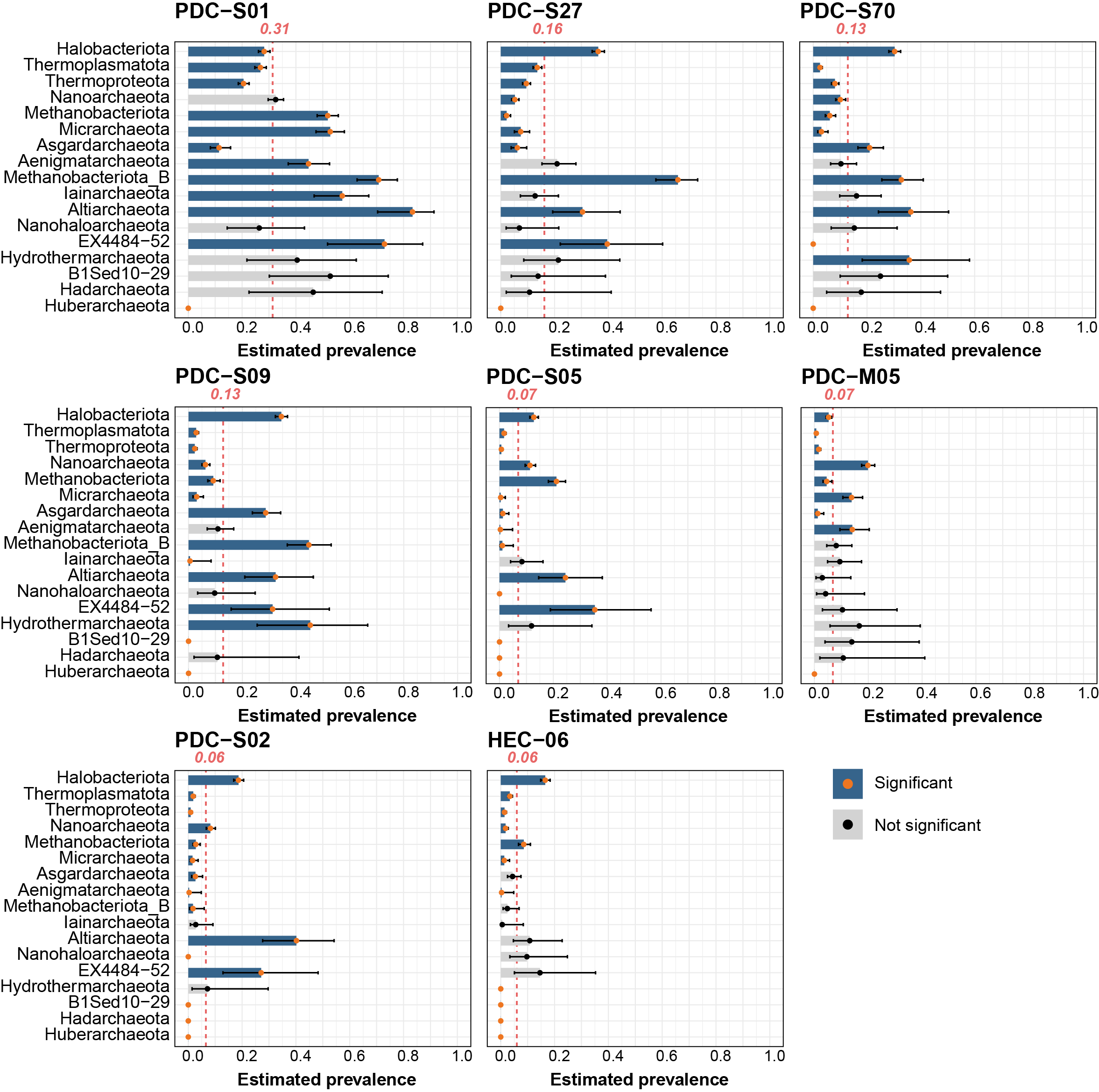


**Supplementary Figure 7. Taxonomic distribution of the archaeal PDC-core defensome.** PDC systems in the top 20 most prevalent archaeal antiviral systems. The bar charts represent the percentage of genomes in each phylum containing the defense system. The red line indicates the average prevalence of each defense system across all archaeal genomes. Blue bars indicate a significant difference in the prevalence of a system against the overall archaeal mean. Error bars indicate confidence intervals.


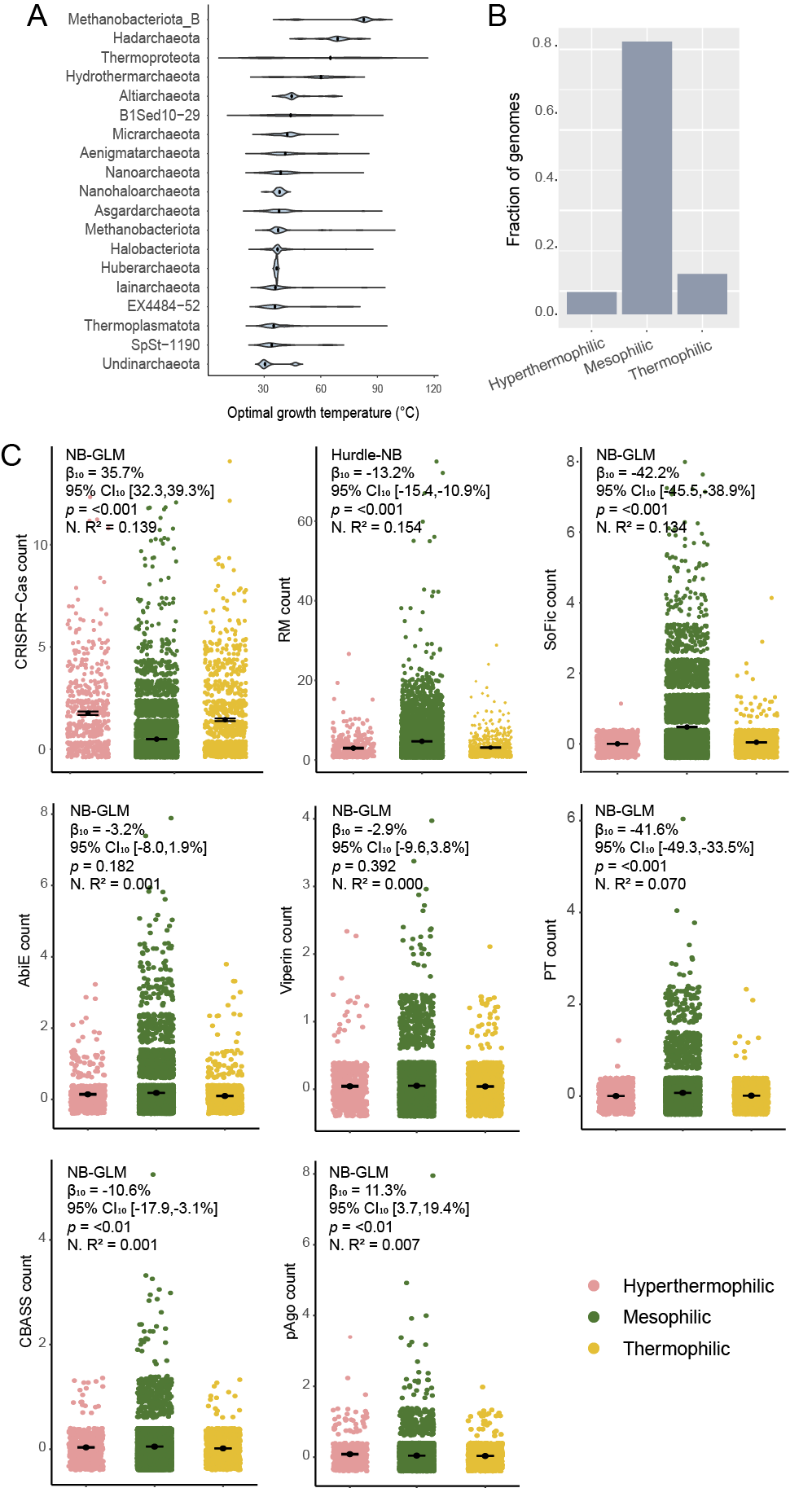


**Supplementary Figure 8. Optimal growth temperature (OGT) is associated with variation in archaeal Cas counts.** **A)** Violin plots of optimal growth temperature per genome across archaeal phyla. Optimal growth temperature was calculated using the Tome algorithm as described in Methods. **B)** Bar plot of the fraction of genomes in the archaeal dataset classified as mesophilic (OGT = 25 to <50 °C), thermophilic (OGT = ≥50 to <80) or hyperthermophilic (OGT = ≥80 °C), following standard definitions of these temperature ranges. **C)** Jittered dot plots showing system counts per genome across optimal growth temperature categories, with mean + SE error bars. Inset statistics derive from negative binomial generalized linear models (NB-GLM) with temperature as a continuous predictor. Statistics for RM abundances derive from a hurdle-negative binomial model (Hurdle-NB) to account for zero-deflation and overdispersion. B_10_: percent change in expected system abundance per 10 °C increase in optimal growth temperature; 95% CI_10_: 95% confidence interval for B_10_; *p*: Wald test *p*-value; N. R2: Nagelkerke’s pseudo-R^2^. Strong positive associations were observed between system abundance per genome and temperature for Cas systems, whereas strong and negative associations were observed for RM, SoFic and PT, with temperature accounting for 7-15% of the model-based variation in system counts. Significant but weak associations between system abundance per genome and temperature were found for argonaute and CBASS, explaining <1% of the model-based variation. No significant association between system abundance per genome and temperature were found for AbiE or viperin.

**
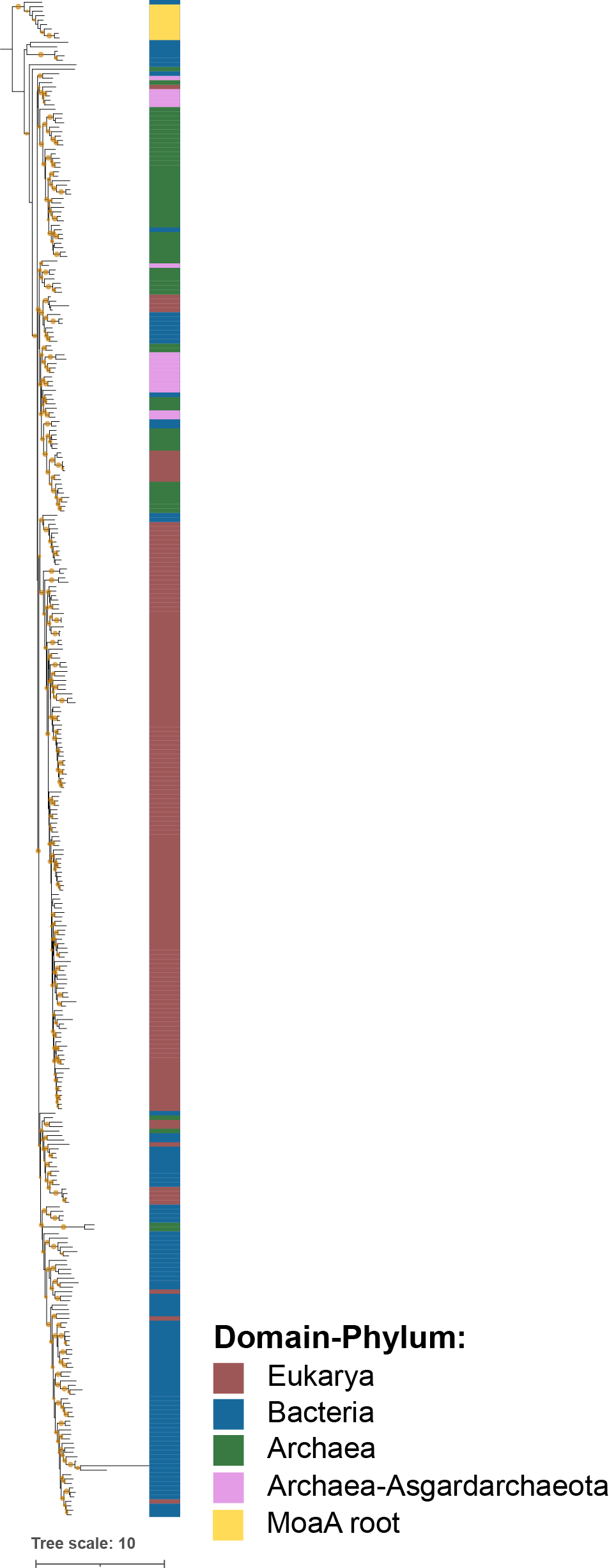
**

**Supplementary Figure 9.** **Phylogenetic analysis of viperins closely related to eukaryotic viperins**. Prokaryotic viperins that clustered near eukaryotic viperins in a preliminary, broad phylogenetic tree (see Methods) were extracted and re-analyzed to investigate the evolutionary origins of eukaryotic viperins. The color strip indicates taxonomic classification: blue for bacteria, red for eukaryotes, green for archaea, and pink for asgard archaea. Bootstrap support values (≥70%) are marked as dots at the corresponding nodes. Tree was rooted using MoaA sequences (yellow).

**
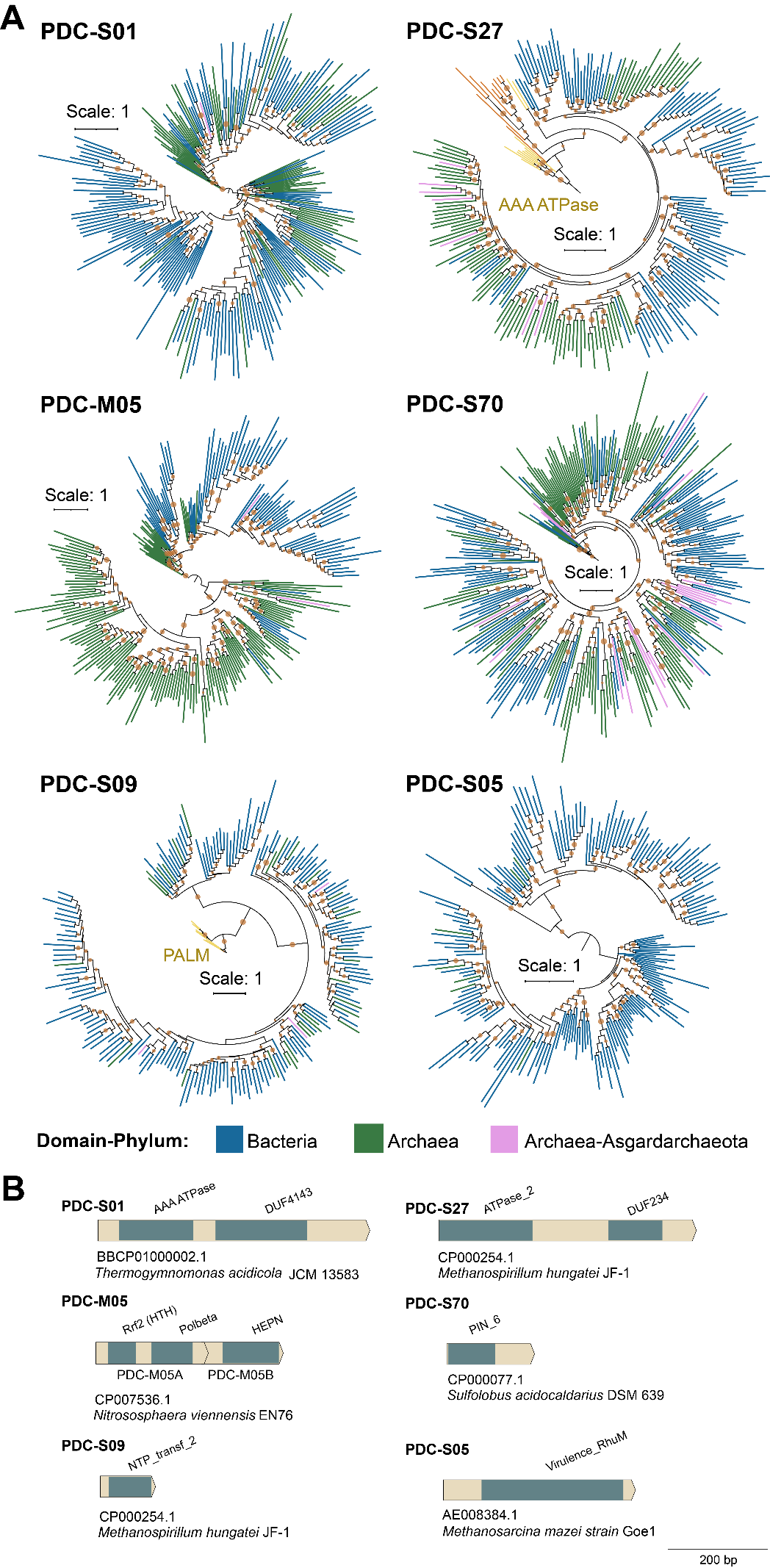
**

**Supplementary Figure 10. Evolutionary origins of the PDC-core defensome**. **A)** Phylogenetic trees of the most prevalent PDC systems in the prokaryotic core-defensome. Branch colors indicate taxonomic classification: blue for bacteria, red for eukaryotes, green for archaea, and pink for asgard archaea. Bootstrap support values (≥70%) are marked as dots at the corresponding nodes. Trees were rooted at midpoint, except PDC-S27 and PDC-S09, which were rooted using AAA ATPases (PF00004), and DNA polymerase beta PALM domain (PF14792) sequences, respectively (yellow). **B)** Examples of representative loci of the putative core defensome. Domain predictions are based on Pfam annotations. ATPase: adenosine triphosphatase; DUF: domain of unknown function; Rfr2: Rfr2 family transcriptional regulator; Polbeta: polymerase beta, nucleotidyltransferase; HEPN: higher eukaryotes and prokaryotes nucleotide-binding; PIN: PilT N-terminal; NTP_transf: nucleotidyl transferase.

**OTHER SUPPLEMENTARY DATA**

**Supplementary Table 1. List of genomes analyzed.**

**Supplementary Table 2. Defense systems predicted by Padloc.**

**Supplementary Table 3. Defense systems predicted by Defense Finder.**

**Supplementary Table 4. Cas cassettes predicted by CRISPR-Cas typer in archaeal genomes.**

**Supplementary Table 5. CRISPR loci predicted by CRISPR-Cas typer in archaeal genomes.**

**Supplementary Table 6. Prediction of restriction-modification systems in Archaea using the REBASE-approach.**

**Supplementary Table 7. List of defense systems identified by Padloc and DefenseFinder.**

**Supplementary Table 8. Comparison of Padloc and DefenseFinder performance by clustering of predicted defense system proteins.**

**Supplementary Table 9. Statistical comparison of the core defensome prevalence across archaeal phyla.**

**Supplementary Table 10. Amino acid sequences used for phylogenetic analysis.**

**Supplementary Data 1. Phylogenetic trees for Fig. 5, Fig. S9 and Fig. S10.**
